# Efficacy Assessment of an Uncharged Reactivator of NOP-Inhibited Acetylcholinesterase Based on Tetrahydroacridine Pyridine-Aldoxime Hybrid in Mouse Compared to Pralidoxime

**DOI:** 10.3390/biom10060858

**Published:** 2020-06-04

**Authors:** André-Guilhem Calas, Anne-Sophie Hanak, Nina Jaffré, Aurélie Nervo, José Dias, Catherine Rousseau, Charlotte Courageux, Xavier Brazzolotto, Pascal Villa, Adeline Obrecht, Jean-François Goossens, Christophe Landry, Johan Hachani, Fabien Gosselet, Marie-Pierre Dehouck, Jagadeesh Yerri, Maria Kliachyna, Rachid Baati, Florian Nachon

**Affiliations:** 1 Département de Toxicologie et Risques Chimiques, Institut de Recherche Biomédicale des Armées, F-91220 Brétigny-sur-Orge, France; anne-sophie.hanak@def.gouv.fr (A.-S.H.); nina.jaffre@def.gouv.fr (N.J.); aurelie.nervo@def.gouv.fr (A.N.); jose.dias@def.gouv.fr (J.D.); catherine.delage@def.gouv.fr (C.R.); charlotte.courageux@def.gouv.fr (C.C.); xavier.brazzolotto@def.gouv.fr (X.B.); florian.nachon@def.gouv.fr (F.N.); 2 COGNition and ACtion Group, UMR 8257, CNRS-MD-UPV, Centre universitaire des Saints-Pères, F-75006 Paris, France; 3 CNRS, Université de Strasbourg, PCBIS Plate-forme de Chimie Biologique Intégrative de Strasbourg UMS 3286, F-67412 Illkirch, France; pvilla@unistra.fr (P.V.); aobrecht@unistra.fr (A.O.); 4 Labex MEDALIS, F-67000 Strasbourg, France; 5 Université de Lille, ULR-7365—GRITA Groupe de Recherche sur les Formes Injectables et Technologies Associées, F-59000, Lille, France; jean-francois.goossens@univ-lille2.fr; 6 Université d’Artois (UArtois), UR 2465, LBHE Laboratoire de la Barrière Hémato-Encéphalique, F-62307 Lens, France; christophe.landry@univ-artois.fr (C.L.); johan.hachani@univ-artois.fr (J.H.); fabien.gosselet@univ-artois.fr (F.G.); mpierre.dehouck@univ-artois.fr (M.-P.D.); 7 UMR CNRS 7515, ICPEES Institut de Chimie et Procédés pour l’Énergie, l’Environnement et la Santé, F-67087 Strasbourg, France; jagadeesh.yerri@gmail.com (J.Y.); kliachyna.maria@gmail.com (M.K.); rachid.baati@unistra.fr (R.B.)

**Keywords:** organophosphorus nerve agents, oxime, cholinesterase, reactivation, ventilation, pharmacodynamics, blood-brain barrier crossing

## Abstract

(1) Background: Human exposure to organophosphorus compounds employed as pesticides or as chemical warfare agents induces deleterious effects due to cholinesterase inhibition. One therapeutic approach is the reactivation of inhibited acetylcholinesterase by oximes. While currently available oximes are unable to reach the central nervous system to reactivate cholinesterases or to display a wide spectrum of action against the variety of organophosphorus compounds, we aim to identify new reactivators without such drawbacks. (2) Methods: This study gathers an exhaustive work to assess in vitro and in vivo efficacy, and toxicity of a hybrid tetrahydroacridine pyridinaldoxime reactivator, KM297, compared to pralidoxime. (3) Results: Blood–brain barrier crossing assay carried out on a human in vitro model established that KM297 has an endothelial permeability coefficient twice that of pralidoxime. It also presents higher cytotoxicity, particularly on bone marrow-derived cells. Its strong cholinesterase inhibition potency seems to be correlated to its low protective efficacy in mice exposed to paraoxon. Ventilatory monitoring of KM297-treated mice by double-chamber plethysmography shows toxic effects at the selected therapeutic dose. This breathing assessment could help define the No Observed Adverse Effect Level (NOAEL) dose of new oximes which would have a maximum therapeutic effect without any toxic side effects.

## 1. Introduction

The organophosphorus compounds (OP) family includes molecules like parathion used as pesticides, plasticizer, flame retardants, anti-wear additives in jet engine lubricants such as tricresyl phosphate (TCP), as well as highly toxic chemical warfare nerve agents such as VX or sarin. Each year, self-poisonings by OP insecticides lead to around 100,000 deaths worldwide [1]. Recently, attacks were perpetrated with nerve agents in Syria (2013 and 2017) [2,3], Malaysia (2017) [4], and also in the United Kingdom (2018) [5]. The toxic action of these agents is characterized by symptoms such as headache, nausea with vomiting and/or diarrhea, fasciculations progressing to paralysis, loss of consciousness, seizures, and apnea [6]; eventually leading to death. These toxic effects come from the irreversible inhibition of primarily acetylcholinesterase (AChE, E.C. 3.1.1.7) and butyrylcholinesterase (BChE, E.C. 3.1.1.8). Their inhibition results in high concentrations of the acetylcholine neurotransmitter, which leads to the over-stimulation and/or the desensitization of cholinergic receptors located in the synaptic cleft of peripheral and central synapses. 

AChE irreversibly inhibited by OP can be reactivated by oximes reactivators (for a review see [7]). Such reactivators associated with an antimuscarinic drug (atropine) and an anticonvulsant (diazepam) form the current emergency treatment in the event of OP poisoning. However, currently marketed oximes do not readily cross the blood–brain barrier (BBB) due to their permanent positive charge. They are, consequently, poorly active in the central nervous system (CNS). Moreover, despite 60 years of research, none of them display an extensive broad spectrum of reactivation [8].

**Figure 1:**
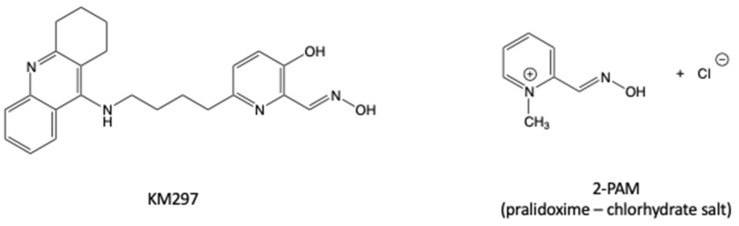
Chemical structures of the oximes used in this study, the reference oxime 2-PAM, and the novel α-hydroxypyridine oxime KM297.

Here we present a toxicological study on human cell lines and a comparative in vivo study in Swiss mice of pralidoxime (2-PAM) with a hybrid tetrahydroacridine pyridinaldoxime reactivator, KM297 (Figure 1), designed to readily cross the BBB and which has shown promising reactivation properties in vitro [9]. 

Blood clearance of 2-PAM and KM297 as a free base and as various salts are monitored by an established pharmacodynamic method and blood AChE and BChE activity is followed after KM297 chlorhydrate (HCl) injection. Cytotoxicity of 2-PAM and KM297 HCl is assessed on 6 different cell lines. The penetration of both oximes through the BBB is measured using a human in vitro model named brain-like endothelial cells (BLECs) [10]: briefly, their cell toxicity is evaluated using a monolayer integrity marker followed by a permeability test on the BLEC monolayer. Protective efficacy of the oximes without atropine co-treatment is assessed in vivo by 24 h-long-surviving tests in VX-challenged and paraoxon-challenged mice using the up-and-down method; paraoxon being the active and toxic metabolite of parathion. Finally, the toxicity of this novel reactivator is assessed by following the potential of KM297 HCl to alter the breathing pattern of mice.

## 2. Materials and Methods 

### 2.1. Animals

We used 8- to 10-week-old male Swiss mice (Janvier, Le Genest-Saint-Isle, France), weighing 37–43 g at the experimentation time. The animals (3–4/cage) were housed for 7–14 days before the experiments in an environment maintained at 23 °C with controlled humidity on a 12 h dark/light cycle with light provided between 7 a.m. and 7 p.m. They were given food and tap water ad libitum. All experiments were carried out in compliance with the European Directive on the protection of animals used for scientific purposes (2010/63/UE) and were approved by our Institutional Animal Care and Research Advisory Committees (2012/22.0 and 12-98). 

### 2.2. Chemicals

VX was obtained from DGA maîtrise NRBC (Vert le Petit, France), 2-PAM from Pharmacie Centrale des Armées (Orléans, France), KM297 from UMR CNRS 7515 ICPEES (Strasbourg, France). Heparine, Cremophor-EL, DMSO, iso-OMPA, DTNB, acetylthiocholine, and butyrylthiocholine were purchased from Sigma-Aldrich.

Excluding the cytotoxic study, 2-PAM (chlorhydrate salt) was directly dissolved into physiological serum (0.9% NaCl) to obtain the desired concentration (see protocol of each experiment). KM297 free base was first dissolved into DMSO. The solution was then 10-fold-diluted into Cremophor-EL with a final 10-fold dilution into 0.9% NaCl to obtain the final concentration of 1 % *v*/*v* DMSO (see protocol of each experiment). The three different salts of KM297, namely chlorhydrate (KM297 HCl), acetate (KM297 AcOH) and methylsulfonate (KM297 MSA) were directly dissolved into 0.9 % saline solution apart from cytotoxic study. 

### 2.3. Inhibition of AChE by OP Agents

#### 2.3.1. Inhibition of Human AChE (hAChE) by VX

Recombinant hAChE was produced and purified as previously described [11]. The stock solution of VX was 5 mM in isopropanol. Inhibition of purified hAChE at 120 µM [12] was achieved with a 5-fold excess of VX (10-fold for standard reactivation curves) in sodium phosphate buffer (0.1 M, pH 7.4, 0.1% BSA) at 25 °C. Complete inhibition of hAChE was monitored by measuring the residual activity as previously described [13]. After a 20 min-incubation, excess VX was removed by desalting on a PD-10 column (GE Healthcare, Uppsala, Sweden) in the same sodium phosphate buffer.

#### 2.3.2. Inhibition of Murine AChE (mAChE) by Paraoxon

Recombinant mAChE was produced and purified as hAChE. The stock solution of paraoxon was 100 µM in DMSO. Inhibition of purified mAChE at 113 µM [14] was achieved with a 10-fold excess of paraoxon in sodium phosphate buffer (0.1 M, pH 7.4, 0.1% BSA) at 25 °C. Complete inhibition of mAChE was monitored by measuring the residual activity as previously described [13]. After a 15 min-incubation, excess paraoxon was removed by desalting on a PD-10 column (GE Healthcare) in the same sodium phosphate buffer.

### 2.4. Plasma Test Protocols

#### 2.4.1. Monitoring of the reactivability of blood samples 

This protocol has been previously fully described [15]. 

##### Standard Curves of Reactivation

Briefly, VX-inhibited hAChE or paraoxon-inhibited mAChE solution in sodium phosphate buffer was incubated for 30 min at 37 °C in the presence of different concentrations of oximes diluted in heat-inactivated mice plasma (0, 1, 5, 10, 50, 100, 150, and 250 µM). Ten-microliter aliquots of each sample were transferred to cuvettes containing 1 mM acetylthiocholine in 1 mL Ellman’s buffer (sodium phosphate 0.1 M, pH 7.4, 0.5 mM DTNB, 25 °C) for monitoring AChE activity at 412 nm. The percentage of reactivated enzyme (% E_react_) (either VX-inhibited hAChE or paraoxon-inhibited mAChE) was calculated as the ratio of the recovered OP-inhibited AChE activity and HI-6 reactivated hAChE activity, which is considered as maximal reactivation (i.e., % E_react_ = 100%). HI-6 reactivation was achieved by incubating VX-inhibited hAChE with 200 µM HI-6 diluted in heat-inactivated plasma. Three independent experiments were carried out for each oxime.

Standard curves were fitted with GraphPad Prism software using a standard asymptotic/exponential time-dependent reactivation equation giving % E_react_ in the function of the oxime concentration ([Ox]):

(1) $$\%\;E_{react}=100\times \left(1-e^{-k_{r2}\times \left[Ox\right]\times t}\right)\frac{IC_{50}^{n}}{IC_{50}^{n}+{\left[Ox\right]}^{n}}$$

where k_r2_ is the apparent bimolecular rate constant (µM^-1^.min^-1^) and t = 30 min for these series of experiments, IC_50_ = 0.25 µM and n, the Hill coefficient. 

##### Blood Sampling

At least 24 h before the experiment, mice were anesthetized with isoflurane gas (Vetflurane^®^, Virbac, France) allowing their hind limbs to be shaved using a 3-min-long application of commercial depilatory cream. Mice were returned to their cages to allow recovery and complete anesthesia washout. On the day of experimentation, mice received intraperitoneal (i.p.) injection of oxime at 23.3 µmol/kg, which corresponds to the highest dose of the soluble free base or 100 µmol/kg analogous with our previous studies [15]. At various time-points (0, 2, 5, 10, 15, 30, 60, and 180 min after oxime injection), the saphenous vein was drilled with a needle and approximately 15 µL of blood was collected with a heparinized capillary tube and deposited in a collection tube containing 2 µL of sodium heparin (Choay^®^, Sanofi, France). Plasma was next isolated from erythrocytes by centrifugation at 4 °C, 3000 g for 10 min. Plasma samples were then heated 30 min at 56 °C and treated as previously described in chapter 2.4.1.1. to obtain the percentage of the reactivated enzyme (% E_react_) with a one-compartment model. T_max_ corresponds to the time when the curve reaches a peak of % E_react_. The areas under the percentage of reactivation curve (AUC) and the first-moment curve (AUMC) were calculated using the trapezoidal rule. Mean residence time (MRT) was calculated as the ratio of AUMC to AUC [16].

#### 2.4.2. Follow-up of Endogenous Blood AChE and BChE Activity

As in paragraph 2.4.1.2., mice were prepared for blood collection at the same time-points after KM297 HCl i.p. injection at 100 µmol/kg. Whole blood samples were diluted 100-fold in sodium phosphate buffer (0.1 M, pH 7.4, 0.1% BSA). Eighteen-microliter aliquots of each sample were transferred to a 96-well plate containing 10 mM acetylthiocholine or butyrylthiocholine in Ellman’s buffer with or without iso-OMPA, a BChE inhibitor [17], for a 40-min measurement with a 10 s time-interval at room temperature for AChE or BChE activity respectively.

### 2.5. IC_50_ of Cholinesterase (ChE) Activity by KM297 HCl

IC_50_ is the concentration of KM297 HCl that inhibits half of hAChE, mAChE, or human BChE (hBChE) activity. Recombinant hBChE was produced in eukaryotic cells as described earlier [18] and the protein purified by BChE specific affinity (Hupresin; CHEMFORASE, Rouen, France) followed by size exclusion chromatography (Superdex 200, GE Healthcare), as previously described [19]. Briefly, hAChE, mAChE, or hBChE diluted in sodium phosphate buffer was incubated for 1 min at room temperature with KM297 HCl in Ellman’s buffer. Acetylthiocholine or butyrylthiocholine diluted in Ellman’s buffer was added to the previous solution and the resulting cholinesterase (ChE) activity was immediately measured using a microplate reader (SPARK, Tecan) at 412 nm for 1 min.

### 2.6. Cytotoxicity

#### 2.6.1. Cell Lines

Cell lines were purchased from ATTC (Manassas, VA, USA), grown in their respective media and stored as master cell banks and working cell banks. New aliquots were thawed and grown for each experiment. The following cell lines were used:

HEK293 (ATCC CRL-1573): human kidney embryonic epithelial cells
HeLa (ATCC CCL-2): human cervix epithelioid carcinoma cells
MCF-7 (ATCC HTB-22): human breast adenocarcinoma cells
HepG2 (ATTC HB-8065): human liver hepatocellular cells
Caco-2 (ATCC HTB-37): human colon adenocarcinoma cells
HL-60 (ATCC CCL-240): human promyelocytic leukemia cells

#### 2.6.2. Cell Culture

For HEK293, MCF-7, HeLa and HepG2 cells, EMEM (Sigma, St-Quentin Falavier, France, M5650) was used supplemented with 10 % FBS (Invitrogen 10270-106) and the following compounds: 2 mM L-glutamine (PAA-GE-Healthcare, Velizy-Villacoublay, France M11-004), 100 U/mL penicillin/100 µg/mL streptomycin (Sigma P0781) and 1 mM sodium pyruvate (Invitrogen-Fisher Scientific, Illkirch, France 11360-039). The same medium was employed for Caco-2 cells, but with an increase to 20% for the FBS (Invitrogen 10270-106).

HL-60 cells were grown in Iscove’s Modified Dulbecco’s Medium (Sigma 51471C) with 20% FBS (Invitrogen 10270-106) and 100 U/mL penicillin/100 µg/mL streptomycin (Sigma P0781).

Cells were seeded at different concentrations into microtiter plates (Greiner Bio-One, Courtaboeuf, France, 655090) according to cell type using automated multidrop seeder (Thermo Fisher Scientific, Courtaboeuf, France): 5 × 10^4^ cells/well for HeLa and Caco-2 cells, 10^5^ cells/well for HEK293 cells, 2 × 10^5^ cells/well for MCF-7 and HepG2 cells, 2.5 × 10^5^ cells/well for HL-60 cells in a volume of 50 µL/well.

#### 2.6.3. Treatment 

KM297 HCl and 2-PAM were dissolved in DMSO at a final concentration of 0.1 M. Aliquots were prepared and frozen at −20 °C before use. An internal control of cytotoxicity with 50 µM chlorpromazine (Sigma C8138) was included in all experiments [20]. After 24 h of incubation (cell incubator, 37 °C, 5% CO_2_), cells were treated by adding 50 µL of medium containing KM297 HCl or 2-PAM (or controls) at twice the final concentration. The dose-response curves were built using the following final concentrations: 100, 30, 10, 3, 1, and 0.3 µM. All treatments were done using a fully automated robotized platform (Beckman Coulter, Villepinte, France). DMSO concentration was the same in all wells (0.5% final).

#### 2.6.4. Cell Viability Assay

Cell viability assays were performed 48 h after treatment. Cell viability was measured using the WST-1 assay (Ozyme, Saint-Quentin-en-Yvelines, France) according to the manufacturer’s protocol as described by Houel et al. [21]. Briefly, WST-1-containing medium was added to cells and cell viability was determined by measuring absorbance at 450 nm using an Envision reader (Perkin Elmer, Villebon-sur-Yvette, France) 1 h after incubating at 37 °C and 5% CO_2_. Each measurement was performed in triplicate and results were expressed as means of three independent experiments.

Statistical calculation of Z’ [22] was performed in all experiments. A Z’ > 0.5 confirms that the results were significant and the assay is robust.

(2) $$Z^{\prime }=1-3\times \frac{\left(SD_{+}+SD_{-}\right)}{\left(Mean_{+}-Mean_{-}\right)}$$

where $SD_{+}\;$= standard deviation of DMSO-treated cells, $SD_{-}$ = standard deviation of cytotoxic active control (chlorpromazine), $Mean_{+}$ = mean of DMSO-treated cells, $Mean_{-}$ = mean of cytotoxic active control.

### 2.7. Transport Experiments Across the Human in vitro BBB Model

#### 2.7.1. Design of the Human In Vitro BBB Model

The in vitro model was set up using stem cells as described previously [10]. Endothelial cells were derived from human umbilical cord blood CD34^+^-cells. In accordance with French legislation, the infant’s parents signed informed consent. The collection protocol was approved by the French Ministry of Higher Education and Research (CODECOH number DC2011-1321). 

Briefly, CD34^+^-hematopoietic stem cells were isolated and differentiated into endothelial cells as described by Pedroso et al., [23]. Then these endothelial cells were seeded onto matrigel (BD Biosciences, Franklin Lakes, NJ, USA, 354230) coated Transwell inserts (Corning, NY, USA, 3401). Endothelial cells (8 × 10^4^ cells/cm^2^) were seeded onto the insert and cultured in the presence of 5 × 10^4^ cells/cm^2^ bovine brain pericytes seeded at bottom of a well (12-well format). The medium of this co-culture [ECM-5 composed of ECM basal medium (Sciencell, Carlsbad, CA, USA) supplemented with 5% (*v*/*v*) fetal calf serum, 1% (*v*/*v*) EC growth supplement (Sciencell) and 50 µg/mL gentamycin (Biochrom AG, Berlin, Germany)] was renewed every 2 days. After 6 days of co-culture with pericytes, the endothelial cells differentiated into BBB endothelial cells and were ready for experiments. This model named BLECs reproduces several features of the in vivo BBB [24,25,26]. 

#### 2.7.2. Endothelial Permeability Measurement

Endothelial permeability coefficients were determined as described by Dehouck et al., [27]. The inserts, containing an endothelial cell monolayer or only coated with matrigel, were transferred into 12-well plates containing 1.5 mL of Ringer-HEPES (RH) buffer saline (150 mM NaCl, 5.2 mM KCl, 2.2 mM CaCl_2_, 0.2 mM MgCl_2_6H_2_O, 0.6 mM NaHCO_3_, 5 mM HEPES, pH 7.4) per well, thus constituting the abluminal compartment. Then, 0.5 mL of RH buffer saline containing 5 µM or 50 µM of the compound to be tested (2-PAM, KM297 HCl or diazepam, which easily crosses the BBB and was used as the positive control) was added to the upper (luminal) compartment, in place of the culture medium in contact with the BBB endothelial cells. After different time-points (20, 40, and 60 min), filters were transferred into a new well containing RH buffer saline. All incubations were performed at 37 °C. After 60 min, the concentration of the molecules tested was determined in the upper and lower compartments.

To assess the toxicity of the molecules on the BLECs, Lucifer yellow (LY) (Sigma-Aldrich, L0259) that poorly crosses the BBB, was used as the monolayer integrity marker. Diffusion of 50 µM LY was tested alone (control) or incubated with KM297 HCl or 2-PAM, as described above. At 60 min, a 200 µL aliquot from a lower compartment at each time point and a 20 µL aliquot of the initial solution placed in the upper compartment were put in a fluorimeter (Synergy H1; BioTek, Winooski, VT, USA) for LY concentration measurements or a liquid scintillation counter (Perkin Elmer, Courtabœuf, France) for ^3^H-diazepam measurements, while the concentrations of 2-PAM and KM297 HCl were determined by mass-spectrometry in the solutions from the upper and lower compartments [28].

The endothelial permeability coefficients (Pe) to LY, 2-PAM, KM297 HCl, and diazepam were calculated using the clearance principle to generate a concentration-independent parameter as described by Siflinger-Birnboim et al., [29]. First, the volumes cleared were plotted versus time (20, 40, 60 min) and the slopes were estimated by linear regression for both insert only coated with matrigel (PSf) and for insert coated with matrigel and seeded with cells (PSt). PSe was calculated according to the following formula: 1/PSe = 1/PSt-1/PSf where "S" represented the surface area of the porous membrane of the insert. PSe was divided by the membrane surface (1.12 cm^2^) to generate the endothelial permeability coefficients (Pe) to the studied molecules expressed in cm/min.

#### 2.7.3. LC-MS/MS Analysis

The molecules (2-PAM and KM297 HCl) were analyzed by an LC-MS/MS system consisted of a Thermo Scientific™ TSQ Vantage™ Triple Quadrupole mass spectrometer outfitted with a Thermo Accela™ LC System. The separation was carried out on a Synergi™ Hydro-RP 80 Å reverse phase column (3 × 150 mm, particle size 4 μm) in isocratic mode using a mixture of water (A)/acetonitrile (B), both solutions containing 0.1% formic acid. The mobile phase was delivered through the column (temperature maintained at 35 °C) at a flow rate of 0.7 mL/min, whereas the temperature of the autosampler was kept at 8 °C with an injection volume of 10 μL. Chromatographic conditions are summarized in Table 1. The detection was performed in liquid chromatography-tandem mass spectrometry with a source operating in positive ion mode. The mass spectrometer was used in the multiple reaction monitoring mode (MRM). The precursor/product ion transitions with the highest intensity and/or specificity were selected to give the maximum sensitivity and selectivity for each analyte (Table 2). The acquired MRM data were processed and quantified with Xcalibur™ software. Each experiment was repeated at least three times.

### 2.8. LD_50_ Estimation and Protective Index Using the Up-and-Down Method

LD_50_ was estimated using the improved method of Dixon’s up-and-down procedure described by Rispin et al., [30]. This method uses an iterative dose-selection algorithm. It consists of a single ordered dose progression in which mice are dosed, one at a time, at 24 h intervals. The first animal receives a dose a step below the level of the best estimate of the LD_50_. If the mouse survives, the dose for the next animal has increased by 1.1-fold the original dose; if it dies, the dose for the next animal is decreased by the same factor. In our particular conditions, the testing stops when one of the following criteria is met: (1) three consecutive animals survive at the highest dose (which is normally 2000 mg/kg); (2) five reversals occur in any six consecutive animals tested; (3) at least four animals have followed the first reversal and the specified likelihood-ratios which compare the maximum likelihood estimate for LD_50_ with LD_50_ values above and below exceed the critical value of 2.5. Profile likelihood methods are used to estimate confidence intervals. In practice, the stopping criteria, the resulting LD_50,_ and the corresponding confidence interval were determined using the AOT 425 Pgm software as recommended by OECD [31]. The antidotal efficacy of the oximes is expressed as a protective index (PI) with 95% confidence interval. The PI corresponds to the ratio of LD_50_ of the studied OP agent (either VX or paraoxon) combined with oxime treatment on LD_50_ of OP alone. LD_50_(VX) was previously established with a value of 15.4 µg/kg [15].

### 2.9. Ventilatory Effect Measurement Using Double-Chamber Plethysmography

Ventilatory parameters were recorded in a double-chamber plethysmograph (DCP, EMKA Technologies, Paris, France) according to the method described and validated by Nervo et al., [32]. Briefly, this system consists of two interconnected Plexiglas chambers, the head chamber, and the body chamber. The awake mouse is maintained in a conical tube with a small opening to allow the animal’s muzzle to pass through. This restrainer is placed into the body chamber, the mouse’s muzzle being in the head chamber so that there is no airflow between the two compartments. Each chamber is equipped with a pneumotachograph and a differential pressure transducer. The pneumotachograph ensures the air passages with the outside, while the differential pressure transducer measures the pressure differences between the atmosphere and the relevant compartment. There is a linear relationship between airflow and pressure difference. The mouse breathing leads to two distinct air flows which are simultaneously recorded: i-the nasal flow generated by the airflow in and out of the muzzle; and ii-the thoracic flow generated by compression and expansion of air when the thorax rises and falls. Both chambers are connected to a ventilation pump that removes carbon dioxide and moisture formed inside the chamber. 

On the day of the experiment, DCP calibration was performed on two points, high value and a low value by injection of 20 mL and 0 mL of air (corresponding to no flow), respectively. The range of measures had to be included between ± 280 and ± 420 mL/s. The mouse was then placed in the device for a 5–10 min habituation session. Once its spontaneous breathing was calm and regular, the recording was started. The inspiratory time, the expiratory time, the tidal volume, the peak inspiratory flow, the peak expiratory flow, the duration of end-inspiratory pauses and end-expiratory pauses, and the specific airway resistance were continuously measured for 45–50 min using Iox^®^ software (EMKA Technologies, Paris, France) and stored on a computer for subsequent analysis and calculation of additional parameters including the breathing frequency and the minute volume. The first 15 min of the recording corresponded to the physiological breathing of the mouse and were considered as an internal control. The mouse was then gently removed from the plethysmograph to receive an i.p. injection of KM297 HCl and replaced thereafter in the device for the 30-min remaining measurements. Since two experiments were performed in parallel, the KM297 HCl administration was delayed by 5 min between the first and the second mouse. 

We adapted the up-and-down method previously described in chapter 2.8 to determine the KM297 HCl dose considered as the “no observed adverse effect level” (NOAEL). Thus, the first group of mice received 100 µmol/kg KM297 HCl. A 1.1-fold lower dose was then administered to the next animal. This process was repeated until reaching the dose for which no ventilatory effect was visually observed on the plethysmography traces by the experimenter. At the end of the experiment, the mouse was euthanized by an i.p. lethal injection of sodium pentobarbital (Doléthal^®^, Vétoquinol SA, France). 

### 2.10. Data Analysis 

Results are expressed as mean ± SEM. 

For the cell viability assays: The IC_50_ values were calculated using 4 parametric nonlinear regressions by Prism software (version 6.07, GraphPad Software, Inc., San Diego, CA, USA) from the logarithmic dose-response curve. 

For transport measurements through the BBB model: Statistical comparisons of the endothelial permeability were performed between KM297 HCl and 2-PAM or diazepam by nonparametric Mann-Whitney test. 

For DCP analysis: To study the time-course of ventilatory parameters, the values obtained after KM297 HCl injection were compared to the baseline values at T_-5_–T_0_ time period using Friedman ANOVA followed by multiple comparison tests using Dunn’s correction. Thereafter the analysis was carried out on a 5-min recording period free of movements of the mouse selected at the peak of the ventilatory effects, i.e., between 10 and 15 min after the KM297 HCl injection using LabChart software (ADInstruments, Paris, France). For each ventilatory parameter, the normality of data distribution was verified using the Shapiro–Wilk test. The differences between the KM297 HCl pre-injection and post-injection data were compared using a paired Student’s t-tests in case of Gaussian distribution or non-Gaussian Wilcoxon tests. All tests were performed using Prism version 7.0 (GraphPad Software, Inc., San Diego, CA, USA). 

For all tests, statistical differences were considered significant when *p* < 0.05.

## 3. Results

### 3.1. Pharmacodynamic Measurements

#### 3.1.1. Based on the Reactivability of OP-Inhibited AChE

**Figure 2:**
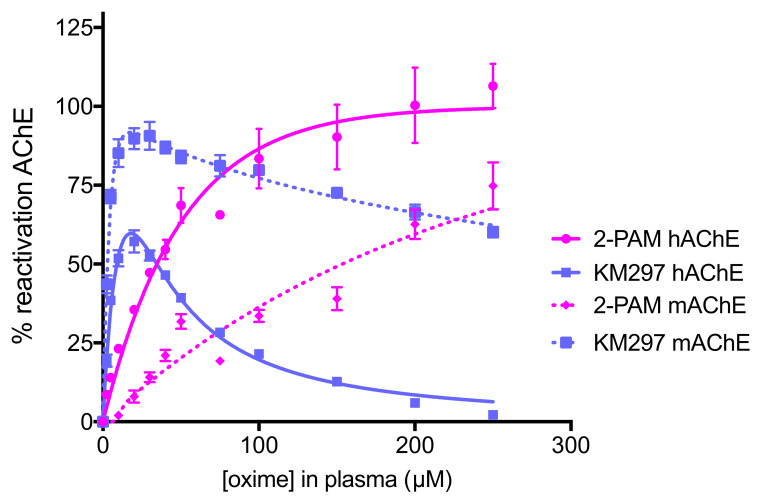
Standard curves of concentration-dependent reactivation of VX-inhibited hAChE and paraoxon-inhibited mAChE by two oximes. VX-inhibited hAChE and paraoxon-inhibited mAChE were incubated with 0–250 µM of oximes (2-PAM or KM297 HCl) diluted in heat-inactivated mice plasma. The recovered activity was determined after 30 min incubation at 37 °C. The best-fit curves and apparent bimolecular rate constants k_r2_ were calculated by nonlinear regression using the equation described in part 2.4.1.1 and GraphPad Prism software. Three independent experiments were performed for each oxime and each inhibited enzyme. Values are presented as mean ± SEM.

Relying on the method previously reported [15], we established standard curves of 2-PAM and KM297 HCl. While a pralidoxime concentration higher than 200 µM allowed to fully reactivate hAChE and increasing pralidoxime concentrations was correlated with increasing mAChE reactivation, KM297 HCl effect on both inhibited hAChE and mAChE was only visible at low concentrations. For concentrations higher than 20 µM, the enzyme reactivation was counterbalanced by a strong inhibition effect (Figure 2). This particular profile of KM297 did not allow us to convert an in situ percentage of AChE reactivation in a unique oxime concentration since a single measured percentage could correspond to two different concentrations, either before or after the reactivation percentage peak. 

**Figure 3:**
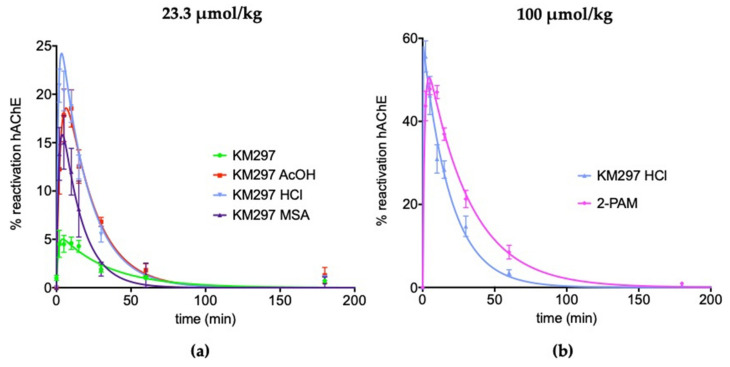
The reactivability of VX-inhibited hAChE by KM297 as free base and salts, and 2-PAM in mice plasma. **(a)** The same dose of 23.3 µmol/kg of KM297 free base and salts: acetate (AcOH), chlorhydrate (HCl) and methylsulfonate (MSA) was administered intraperitoneally to mice (*n* = 8). **(b)** The same dose of 100 µmol/kg of KM297 HCl and 2-PAM was administered intraperitoneally to mice (*n* = 8–10). Blood samples were drawn at various time points (0, 2, 5, 10, 15, 30, 60 and 180 min) after treatment, and the levels of reactivation of VX-inhibited hAChE were determined. Values are presented as percentages of maximum reactivation and points are means ± SEM. Fitting was performed on GraphPad Prism software.

In situ clearance of this reactivator followed by in vitro VX-inhibited hAChE reactivation showed that at the same molar concentration (i.e., 23.3 µmol/kg or 10 mg/kg of KM297 as the free base, which corresponded to its highest concentration completely soluble in 0.9% NaCl) the four galenic forms of the studied oxime presented different pharmacodynamic properties; mainly due to their different reactivation percentage peak. The less soluble free base of the oxime predictably showed the lowest maximal reactivation in mice plasma (React max = 5.1 ± 1.1 %), whereas the chlorhydrate salt showed a higher value (23.0 ± 2.3%) than the two other salts (15.8 ± 2.9% for the methylsulfonate vs. 18.6 ± 2.6% for the acetate). The time to reach the peak of hAChE reactivation in plasma was around 5 min for the different salt forms of KM297 (from 3.5 to 6.5 ± 0.5 min). The last parameter established by the reactivability kinetic profile, the mean residence time (MRT), showed that the hAChE reactivability potential of KM297 salts was eliminated from plasma within 23 min compared to 31.0 ± 3.0 min for the free base (Figure 3a and Table 3). As the KM297 chlorhydrate salt appeared to be the galenic form with the best pharmacodynamic properties, i.e., with the highest hAChE reactivation peak together with a long mean residence time, this salt was selected for the subsequent studies. In particular, its pharmacodynamic profile was compared to pralidoxime at the same therapeutic dose of 100 µmol/kg. In these conditions, KM297 HCl and 2-PAM showed a similar peak of reactivation (57.9 ± 4.3% vs. 50.6 ± 1.7% respectively), but 2-PAM reached its peak later than KM297 HCl (4.5 ± 0.5 min vs. 0.5 min respectively) which was consistent with a longer persistence in mice plasma (30.8 ± 2.8 min vs. 19.5 ± 1.1 min for the MRT respectively) (Figure 3b and Table 3).

#### 3.1.2. Based on Blood AChE and BChE Activities

**Figure 4:**
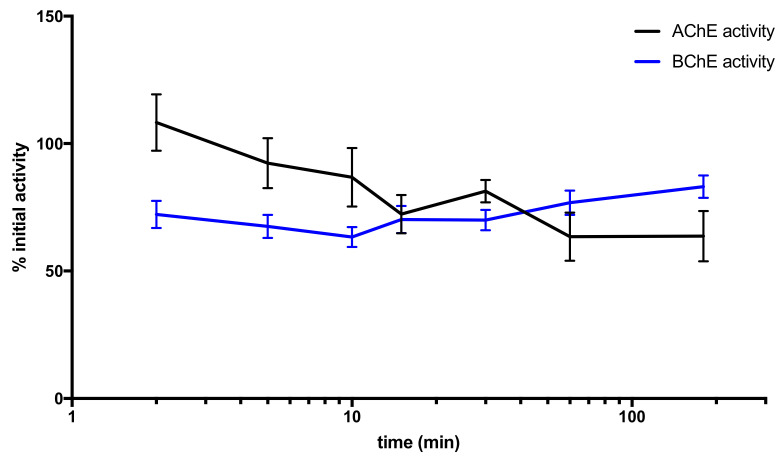
Mice blood AChE and BChE activity follow-up after an i.p. administration of 100 µmol/kg KM297 HCl (*n* = 8–10). Blood samples were drawn at various time points (0, 2, 5, 10, 15, 30, 60, and 180 min) after treatment, and AChE and BChE activity levels were measured. Values are presented as percentages of corresponding initial activity and points are means ± SEM.

The follow-up of mice blood AChE and BChE activities showed a rapid and significant decrease of plasma BChE activity as early as 2 min after KM297 HCl injection that reached a minimum of 63% of the initial activity at 10 min and a slow recovery. Erythrocyte AChE activity decreased more slowly, reached a minimum at 1 h, and remained stable during the 3 h experiment (Figure 4).

### 3.2. IC_50_ Measurements of ChE (hAChE, mAChE, and hBChE) Activity 

**Figure 5:**
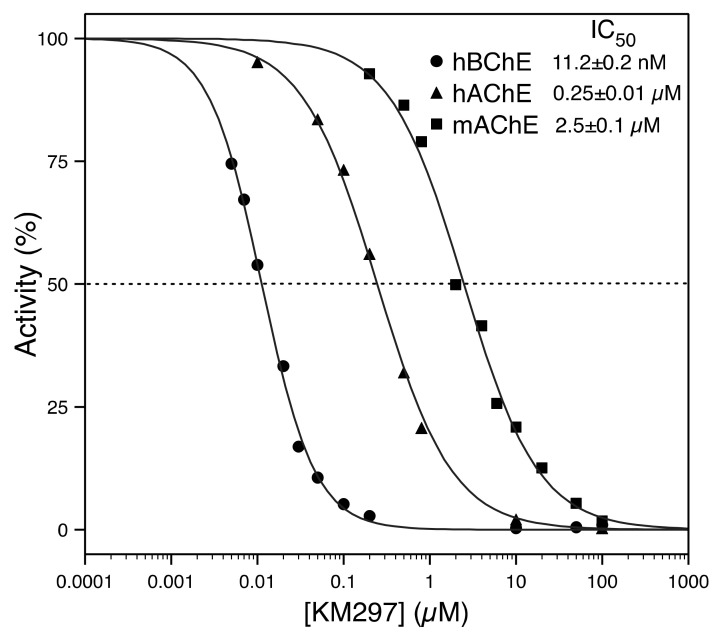
In vitro IC_50_ determination of KM297 HCl for hAChE, mAChE, and hBChE.

An in vitro determination of the half-maximal inhibitory concentration (IC_50_) value of KM297 HCl was performed on recombinant hAChE, mAChE, and hBChE to assess its ChE inhibitor potency. It showed that this oxime induced a 10-fold higher inhibition of hAChE (0.25 ± 0.01 µM) than of mAChE (2.5 ± 0.1 µM). It is also noteworthy that KM297 is a powerful inhibitor of hBChE (11.2 ± 0.2 nM) (Figure 5).

### 3.3. Cytotoxicity

First of all, it is noteworthy that WST-1 assay does not allow to differentiate proliferation decrease and cell viability. However, a cellular confluency-based assay (imaging assay using Incucyte^®^ apparatus from Sartorius) performed in parallel (data not shown) showed that the cells were dying. Thus, we can affirm that, in our study, the WST-1 assay measured a decrease in cell viability.

**Figure 6:**
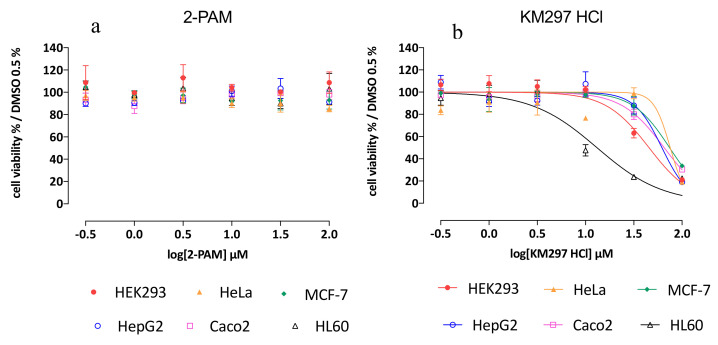
Cell viability WST-1 assay on 6 various cell lines (HEK293, HeLa, MCF-7, HepG2, Caco-2, and HL60 cells) after (**a**) 2-PAM and (**b**) KM297 HCl exposure. All experiments were carried out in triplicate and means ± SEM determined.

While 2-PAM exposure did not induce any deleterious effect on each 6 cell line tested from 0.3 to 100 µM of oxime, KM297 HCl exposure induced a decrease of cell viability on each of them at the dose of 100 µM. Calculated IC_50_ values in each studied cell line are presented in Table 4. It should be noted that each calculated Z’ result reached the ≥ 0.5 value that demonstrates the significance and robustness of the assay. HL60 cells seemed to be the most sensitive to KM297 HCl exposure with an IC_50_ value of 13.1 µM. In the HEK293 cell line, the IC_50_ value was calculated as 45.5 µM while in the other cell lines i.e., HeLa, MCF-7, HepG2, and Caco-2 cells, the IC_50_ was determined to be 75.4, 74.3, 61.9, and 65.3 µM, respectively (Figure 6 and Table 4).

### 3.4. Transport Assessment Across the Human In Vitro BBB Model

Before measuring transportation, the toxicity of 2-PAM and KM297 HCl was tested in the human *in vitro* BBB model. To this extent, the Pe of LY, the small hydrophilic molecule used as BBB integrity marker, incubated with the oximes during the experiment was measured in the presence and absence of oximes. At the dose of 5 and 50 µM, Pe _LY_ values were equivalent to that obtained for LY incubated alone (Pe _LY_ = 0.57 × 10^−3^ ± 0.04 cm/min), confirming the absence of toxicity of these compounds on the endothelial cell monolayer.

**Figure 7:**
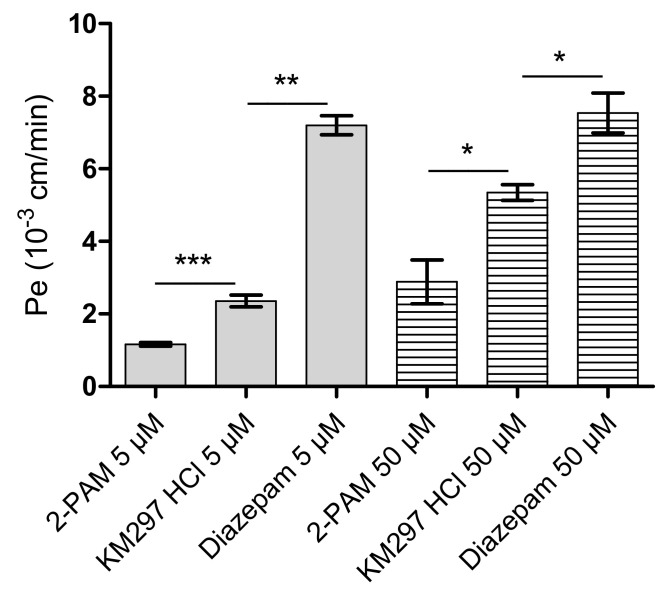
Endothelial permeability coefficient (Pe) of 2-PAM, KM297 HCl, and diazepam measured in the human in vitro BBB model at 5 and 50 µM. Values are presented as mean ± SEM, *n* = 3–12. Comparisons were performed using Mann–Whitney test between KM297 HCl and 2-PAM or diazepam. * *p* < 0.05, ** *p* < 0.01, *** *p* ≤ 0.001).

The passage of 2-PAM and KM297 HCl across BLEC monolayer was assessed using the human in vitro model (Figure 7). The Pe of KM297 HCl was 2- and 1.5-fold greater than that of 2-PAM at the dose of 5 µM and 50 µM, respectively (Pe _KM297, 5 µM_ = 2.36 × 10^−3^ ± 0.49 cm/min vs. Pe _2-PAM, 5 µM_ = 1.16 × 10^−3^ ± 0.18 cm/min, *p* = 0.001 and Pe _KM297, 50 µM_ = 5.35 × 10^−3^ ± 0.53 cm/min vs. Pe _2-PAM, 50 µM_ = 2.89 × 10^−3^ ± 1.81 cm/min, *p* = 0.036). 

As brain penetration of a drug such as diazepam is known to be relatively high, Pe values of KM297 HCl were compared with those of diazepam as a positive control. The Pe of 50 µM KM297 HCl remained significantly lower than that of diazepam (Pe _KM297, 50 µM_ = 5.35 × 10^−3^ ± 0.53 cm/min vs. Pe _diazepam, 50 µM_ = 7.54 × 10^−3^ ± 0.95 cm/min, *p* = 0.024).

Transport of 2-PAM and KM297 HCl across the human in vitro BBB was concentration-dependent at the dose of 5 and 50 µM as endothelial permeability increased with the oximes concentration whereas that of diazepam did not (Table 5).

### 3.5. In Vivo Protective Index Assessment 

Experiments of the up-and-down procedure performed on VX and paraoxon exposure are presented in Table 6 and Table 7, respectively. LD_50_(VX) was already established previously with a value of 15.4 µg/kg [15]. Intraperitoneal injection of the KM297 free base at the dose of 10 mg/kg did not show any protection after the VX challenge as demonstrated by the PI equal to 1 (Table 6). LD_50_ of paraoxon was established to be 796 µg/kg. Alone, 100 µmol/kg of 2-PAM induced higher protective efficacy than the same dose of KM297 HCl (PI value of 2.58 and 1.18, respectively). Intraperitoneal treatment with less than a two-thirds dose of KM297 HCl (i.e., 62 µmol/kg) increased its efficacy after subcutaneous paraoxon challenge (PI = 1.94) (Table 7). LD_50_ of i.p. injection of KM297 HCl was also assessed by the up-and-down method and achieved a value of 300 µmol/kg (Table 8).

### 3.6. Ventilatory Effect Measurements in Mice

Thanks to the accurate monitoring of mice ventilation with the DCP system, a NOAEL dose has been determined. This NOAEL corresponds to the highest i.p. injected dose of KM297 HCl without any observable toxic effects as described below. From the symptomatic dose of 100 µmol/kg, we iteratively decreased the dose of the treatment by the same factor of 1.1. This process was continued down to the dose of 62 µmol/kg for which no ventilatory alteration was visually observed by the experimenter. Six different animals were treated at this dose to confirm the lack of visual effect on breathing. As a result, the observed NOAEL is equivalent to approximately one-fifth of the LD_50_ of KM297 HCl (300 µmol/kg).

**Figure 8:**
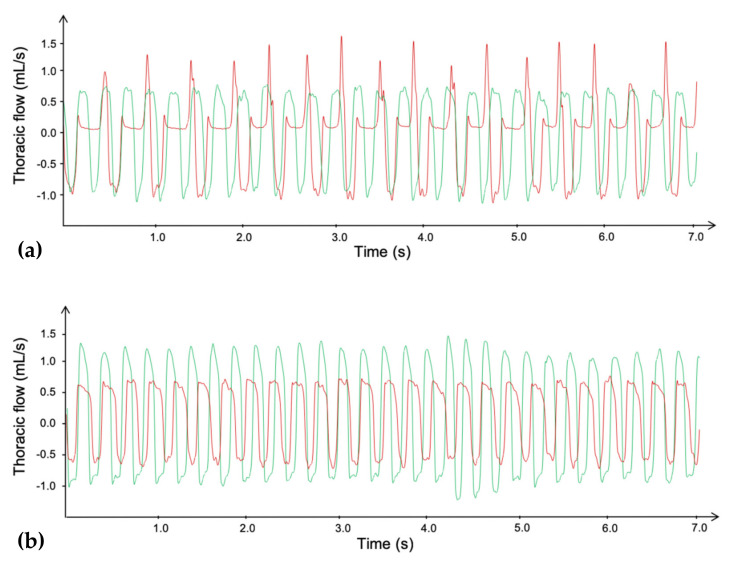
Plethysmography traces of the thoracic flow before (green line) and after (red line) i.p. injection of a dose of 100 µmol/kg **(a)** or 62 µmol/kg **(b)** of KM297 HCl in mice. Traces were selected at the peak of the ventilatory effects, i.e., between 10 and 15 min after the KM297 HCl administration.

**Figure 9:**
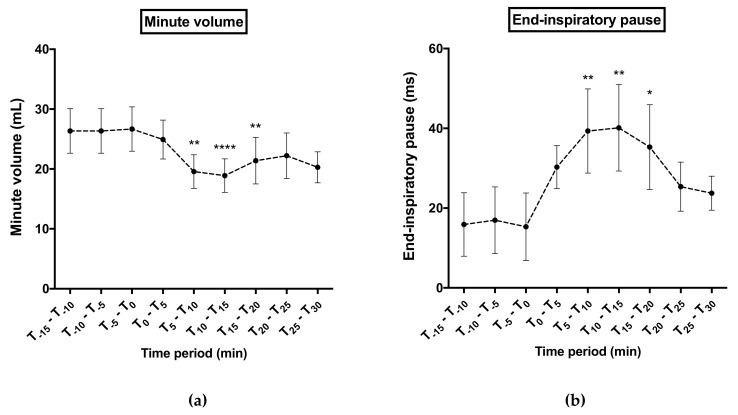
Time course of minute volume (**a**) and end-inspiratory pause (**b**) 15 minutes before 100 µmol/kg KM297 HCl injection up to 30 min later. Results are presented as the mean of 5 min-means of recordings ± SEM, *n* = 10. Comparisons were performed using Friedman ANOVA followed by multiple tests using Dunn’s correction with T_-5_–T_0_ time period considered as the reference period. * *p* < 0.05, ** *p* < 0.01, **** *p* < 0.0001.

Samples of plethysmography recording are presented in Figure 8. For all ventilatory parameters, no significant difference was noticed between both treated groups before KM297 HCl injection (at the dose of 100 µmol/kg and 62 µmol/kg respectively). However, the minute volume was significantly decreased (peak effect: 18.88 ± 2.80 vs. 26.67 ± 3.70 mL, *p* < 0.0001; Figure 9a) probably due to an increase of end-inspiratory pauses duration (peak effect: 40.13 ± 10.85 vs. 15.32 ± 8.48 ms, *p* < 0.01; Figure 9b) for 100 μmol/kg KM297 HCl-treated mice compared to the same mice before treatment, (i.e., at the T_-5_–T_0_ reference period). These ventilatory effects started as early as the 5th minute following KM297 HCl injection, peaked between 10- and 15-min after, for up to 20 min, then gradually decreased over time while remaining present. From these kinetics, the set of ventilatory parameters was then analyzed at the peak of the effects, i.e., at the T_10_–T_15_ time period.

**Figure 10:**
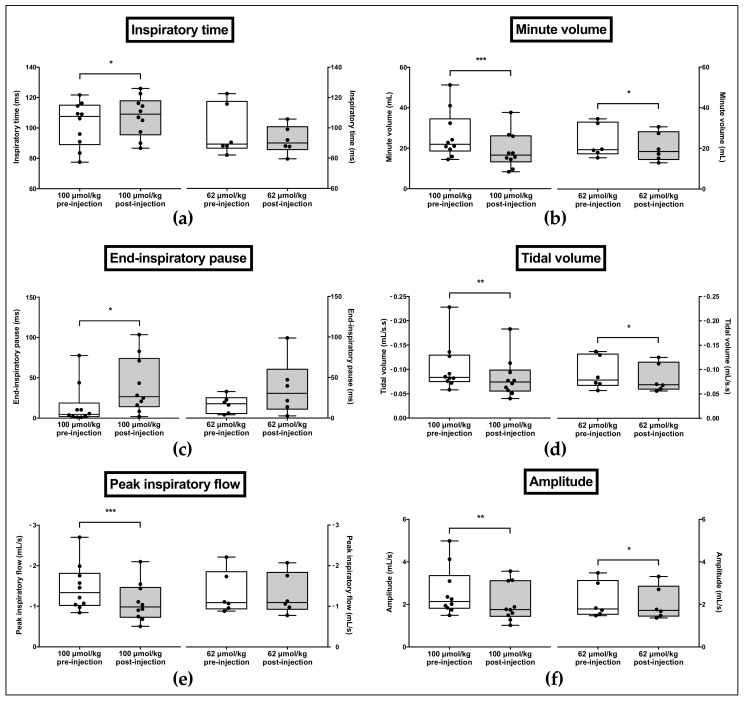
Effects of i.p. administration of 100 µmol/kg or 62 µmol/kg KM297 HCl on ventilatory parameters recorded by double-chamber plethysmography: inspiratory time (**a**), minute volume (**b**), end-inspiratory pause (**c**), tidal volume (**d**), peak inspiratory flow (**e**) and amplitude **(f)**. *n* = 6–10. The line in the middle of the box is plotted at the median. Each individual value is represented as black circles. Whiskers range from the maximum value to minimum value. Comparisons were performed using paired t-tests for normally distributed variables or Wilcoxon tests in case of non-Gaussian distribution. * *p* < 0.05, ** *p* < 0.01, *** *p* < 0.001.

The inspiratory time (*p* = 0.0323, Figure 10a) as well as the end-inspiratory pauses duration (*p* = 0.0371, Figure 10c) were significantly increased following 100 µmol/kg KM297 HCl injection in comparison to pre-injection data, while there was no significant difference following 62 µmol/kg KM297 HCl administration. In contrast, the peak inspiratory flow (*p* = 0.0002, Figure 10e) and the resulting ventilatory amplitude (*p* = 0.0098, Figure 10f) were significantly decreased following 100 µmol/kg KM297 HCl administration, while no difference was observed regarding the peak inspiratory flow after 62 μmol/kg KM297 HCl injection. With this latter dose the ventilatory amplitude remained reduced (*p* = 0.0353), but with a lower intensity than that of the dose of 100 μmol/kg (5.6% vs. 19.8% respectively). A marked reduction in the tidal volume (*p* = 0.0020, Figure 10d) and the resulting minute volume (*p* = 0.0002, Figure 10b) was also noticed following 100 µmol/kg KM297 HCl injection, and persisted with a lower intensity after the administration of the dose of 62 µmol/kg (20.4% vs. 10% respectively for the tidal volume, *p* = 0.0435; 29% vs. 11.4% respectively for the minute volume, *p* = 0.0129). Other ventilatory parameters remained unchanged (data not shown).

## 4. Discussion

KM297 is an uncharged reactivator of OP-inhibited AChE based on tetrahydroacridine pyridine-aldoxime hybrids [9,33]. Its tacrine scaffold has been recently identified as relevant against an OP pesticide poisoning [34]. Designed to cross the BBB, the high lipophilicity of the free base leads to poor solubility [35]: 10 mg/kg (i.e., 23.3 µmol/kg) is the highest achievable concentration in 1% DMSO, 10% Cremophor-EL, 0.9% NaCl. Unfavorable pharmacodynamics of the free base (React-max = 5.1 ± 1.1%) is coherent with an absence of in vivo protection against a VX challenge (PI = 1). This is in agreement with the correlation between pharmacodynamic data and protective efficacy reported earlier [15]. 

New formulations with higher solubility in water such as MSA, AcOH, or HCl salts have been assessed. They present at least 3-fold higher maximal reactivation of VX-inhibited hAChE at their respective t_max_ (React-max value equals to 15.8% ± 2.9%, 18.6% ± 2.6% and 23.0% ± 2.3%, respectively) when injected at the same dose of 23.3 µmol/kg. Due to its more favorable pharmacodynamic profile, the HCl salt was selected for further assessments.

We have shown that the strong reversible inhibition of AChE by KM297 HCl prevents the correlation between pharmacodynamics and pharmacokinetics since one percentage of reactivation could correspond to two oxime concentrations in the standard curve of reactivation. The paraoxon-inhibited mAChE reactivation curve by KM297 HCl presents a similar pharmacodynamic profile which is consistent with the 10-fold higher IC_50_ value measured with mAChE as compared to the one from hAChE. It is worth noting that the 20-fold lower calculated IC_50_ value with hBChE versus hAChE prevents BChE from playing a back-up role in acetylcholine hydrolysis in the case of AChE inhibition. Interestingly, the inhibition property of KM297 could be used as an alternative for reactivation to estimate its plasma concentration. The IC_50_ curve could then allow the correlation of a percentage of inhibition of AChE to a concentration of oxime. 

However, in vivo protective efficacy of KM297 HCl after paraoxon challenge seems to be completely uncorrelated to its maximal percentage of reactivation. Indeed, the same therapeutic dose fixed at 100 µmol/kg KM297 HCl shows a similar pharmacodynamic profile to that of 2-PAM but a different protective index: respectively 1.18 and 2.58. We can reject that this disparity is due to the lack of cerebral effect since our in vitro BBB model established that, as with 2-PAM, KM297 HCl does not affect BBB integrity, though at the same high dose (i.e., 50 µM) KM297 HCl induces cytotoxic effect on several cell lines. This apparent discrepancy can be explained by the huge temporal difference between both observations: endothelial cell monolayer integrity is assessed only 60 min after KM297 HCl exposure while cell line toxicity is measured 48 h post-exposure. Second, this model established that the permeability of KM297 HCl is 2-fold higher than 2-PAM, although lower than the positive control diazepam. Furthermore, 2-PAM and KM297 HCl cross the in vitro BBB in a dose-dependent manner, as already described for 2-PAM using in vivo rat brain microdialysis technique [36].

The potential cytotoxicity and/or systemic toxicity of KM297 could eventually explain its low protective efficacy against OP exposure. Cytotoxicity was evaluated in 6 different human cell lines, each related to different organs: HEK293 to the kidney, HeLa to the uterus, MCF-7 to the breast, HepG2 to the liver, Caco-2 to the colon, HL-60 to the bone marrow. Whereas 2-PAM does not seem to induce any cytotoxicity regardless of the cell line and the concentration of oxime considered, KM297 HCl appears most cytotoxic to HL-60 cells with an IC_50_ of 13.1 µM, followed by HEK293 and then to the four other cell lines: HeLa, HepG2, Caco-2 and MCF-7 cells with similar IC_50_ values greater than 61 µM. By contrast, the IC_50_ of 2-PAM in the HepG2 cell line was 22.8 mM [37]. KM297 HCl appears to be almost 400-fold more cytotoxic than the reference oxime. Bone marrow and consequently blood cells seem to be the most sensitive cells to KM297 HCl. Other tacrine-derived oximes show similar toxicity on HepG2 cells than tacrine [34]. The tacrine scaffold has also been shown, among others, to play a role in hepatotoxicity [38] even if by way of a metabolite [39]. 

We evaluated the systemic toxicity of KM297 HCl through i.p. injection by measuring the LD_50_ at 24 h with the up-and-down procedure. With an LD_50_ at 300 µmol/kg, KM297 HCl is 3-fold more toxic than 2-PAM delivered intramuscularly in the same Swiss male mice (LD_50_(2-PAM) = 1 mmol/kg) [40]. In other words, the therapeutic dose of 100 µmol/kg chosen to compare the protective efficacy of various oximes is 1/3rd of the LD_50_(KM297 HCl) whereas it is only 1/10th of LD_50_(2-PAM).

In addition, mice that received an i.p. injection of KM297 HCl at the dose of 100 µmol/kg showed some characteristic preliminary symptoms related to ChE inhibition such as hypotonia (data not shown). To estimate the level of AChE and BChE inhibition after i.p. injection of KM297 HCl in mice in the absence of OP exposure, we monitored blood AChE and BChE activities. We expected that the time at maximal inhibition of erythrocyte AChE and plasma BChE activities would match that of maximal reactivability seen in the pharmacodynamic curve, as both measurements are proxies of the actual concentration of circulating KM297 HCl. Yet blood AChE activity reached a minima of 60 min after KM297 HCl injection, and plasma BChE activity reached a minima of 10 min after injection, whereas maximal reactivation of VX-inhibited hAChE peaks only 30 s after the injection. However, the dual reactivation/inhibition activity of KM297 HCl could hamper the correct estimation of the time at the peak of reactivation as larger concentrations induce strong inhibition that cancels out the recovery of activity due to reactivation. A longer persistence of this oxime and a delayed peak of concentration of KM297 HCl in the blood compartment could explain this incongruity.

Either method, pharmacodynamics based on reactivability power or inhibition, reports the blood compartment time-evolution of different states of the oxime: bound to ChE for the inhibition method or free oxime for the reactivability method. Consequently, by gathering these results and by taking into account the inhibition potency of KM297 HCl in the reactivability pharmacodynamic data, it is established that this oxime penetrates rapidly into the blood compartment and has a sufficiently low clearance to inhibit a significant portion of circulating AChE and BChE for hours. This long lifetime is corroborated by DCP results, which show a persistence of ventilatory alterations, thought to be related to ChE inhibition (see below), beyond the mean residence time in blood measured as measured by the reactivability method (20 min). Naturally, it must be considered that the DCP results and blood pharmacokinetic data do not show the inhibition of the same compartment i.e., lung vs. blood. Nevertheless, the difference between these two proxy-based estimates of the actual circulating concentration of oximes confirms the main deficiency of the reactivability assay which lies in its inability to take into account the intrinsic inhibition potency of the oxime. In this case, a chemical analytical method to directly measure the concentration of oximes is necessary.

ChE inhibitors such as OP compounds or carbamates may provoke death by respiratory arrest. Breath monitoring with a DCP system confirms that a dose of 100 µmol/kg of KM297 HCl i.p. alters ventilation. In particular, this dose of KM297 HCl induces a significant increase in inspiratory time correlated to the appearance of end-inspiratory pauses. These alterations have previously been established on the breathing of mice challenged with paraoxon and physostigmine, a carbamate inhibitor of AChE [32]. These breathing alterations after administration of a dose of 100 µmol/kg KM297 HCl can be correlated to its potency to inhibit ChE. Indeed, significant differences of minute volume and end-inspiratory pauses duration appear around 10 min after oxime injection, when the inhibition of plasmatic BChE activity is the highest, which means when BChE is no longer able to protect the ventilatory system from the toxic effects of a high circulating acetylcholine concentration [32]. Moreover, we determined the NOAEL at 62 µmol/kg or approximately one-fifth of the LD_50_ of KM297 HCl (300 µmol/kg).

It is more relevant to treat OP-exposed animals with an equitoxic dose of reactivator as reported earlier [41,42], rather than use an equimolar dose of reactivator. But instead of applying an arbitrary factor of LD_50_ to determine the equitoxic dose, we determine the NOAEL dose for each reactivator, based on the alteration of ventilatory parameters relevant to ChE inhibition. Regarding the criteria for establishing the NOAEL dose, even if ventilatory parameters such as inspiratory time and end-inspiratory pause duration increase or peak inspiratory flow decrease are no longer noticeable, other parameter modifications persist such as tidal volume and minute volume and amplitude decrease. The latter are, however, lower than those measured at the dose of 100 µmol/kg. It seems that the visual assessment of ventilation cycle alterations does not appear to be completely accurate in comparison to the calculation of all the different breathing parameters. It seems sufficient to establish a more effective therapeutic dose since there is a significant increase of the protection brought by the KM297 HCl treatment: PI increases from 1.18 at 100 µmol/kg to 1.94 at 62 µmol/kg. A higher protective efficacy with a lower dose has previously been described with other oximes [41]. This result combined with cytotoxic data attests to the narrowness of the therapeutic window of KM297 HCl. 

## 5. Conclusions

We improved the solubility and bioavailability of KM297 with salt derivatives, the most favorable being the chlorhydrate salt. As performed here with KM297 HCl, inhibition potency of oximes could be identified by using the DCP system, which proves its ability to accurately detect minute ventilatory alterations related to ChE inhibition. This system also allows the establishment of a suitable therapeutic dose: we hypothesize that 20% (LD_50_(oxime)) would correspond to its NOAEL value, which should be used as a therapeutic dose in follow-on studies. 

Finally, even if the strong ChE inhibition induced by KM297 HCl should disqualify it as a drug for emergency treatment, its potency in temporarily masking ChE sites from nerve agents, associated with its ability to cross BBB to reach the CNS targets as huperzine does [43], as well as its long-lasting persistence as a reversible adduct with ChE in at least the blood compartment, and of course, its ability to reactivate OP-inhibited ChE could conversely qualify it as an efficient prophylactic treatment for OP nerve agents.

## Figures and Tables

**Table 1:** Chromatographic conditions for LC-MS/MS analysis.

	KM297 HCl	2-PAM	
Column	Synergi Hydro-RP column (4 μm, 3 mm I.D. × 150 mm)		
Mobile phases	A: water + 0.1% formic acid, B: acetonitrile + 0.1% formic acid		
Isocratic mode	A: 35% - B: 65%	A: 99% - B: 1%	
Flow rate	0.7 mL/min		
Injection volume	10 μL		
Total runtime	8 min		

**Table 2:** Multiple reaction monitoring (MRM) transitions and retention times. amu = atomic mass unit.

Compounds	*m*/*z* (amu) Q1	*m*/*z* (amu) Q3 Quantifier	*m*/*z* (amu) Q3 Qualifier	Retention Time (min)	
KM297 HCl	391.3	199.1	373.3	1.5	
2-PAM	137.0	119.1	93.0	2.2	

**Table 3:** MRT: mean residence time, t_max_ and React max respectively x and y coordinates of the peak of reactivability of VX-inhibited hAChE by KM297 as free base and salts, and 2-PAM in mice plasma presented in Figure 3.

Dose (µmol/kg)	Oxime	MRT (min)	t_max_ (min)	React Max (%)
23.3	KM297	31.0 ± 3.0	5.5 ± 1.0	5.1 ± 1.1
23.3	KM297 MSA	15.1 ± 2.7	4.0	15.8 ± 2.9
23.3	KM297 AcOH	22.4 ± 0.2	6.5 ± 0.5	18.6 ± 2.6
23.3	KM297 HCl	20.2 ± 0.5	3.5	23.0 ± 2.3
100	KM297 HCl	19.5 ± 1.1	0.5	57.9 ± 4.3
100	2-PAM	30.8 ± 2.8	4.5 ± 0.5	50.6 ± 1.7

**Table 4:** IC_50_ values of cell viability WST-1 assay, their respective 95% confidence interval and calculated Z’ value following KM297 HCl exposure of each cell line.

Cell Line	IC_50_ (µM)	95 % Confidence Interval	Z’ Value WST-1/ Positive Control Chlorpromazine
HEK293	45.5	[38.0; 54.4]	0.66
HeLa	75.4	[34.4; 165.5]	0.74
MCF-7	74.3	[65.3; 84.4]	0.55
HepG2	61.9	[50.4; 75.9]	0.68
Caco-2	65.3	[58.9; 72.3]	0.51
HL60	13.1	[9.4; 18.4]	0.64

**Table 5:** The statistical comparison performed by a nonparametric Mann–Whitney test of the Pe values at 5 and 50 µM for 2-PAM, KM297 HCl, and diazepam.

2-PAM	*p*-Value
5 µM vs. 50 µM	0.0076 (**)
**KM297 HCl**	
5 µM vs. 50 µM	0.0004 (***)
**Diazepam**	
5 µM vs. 50 µM	0.7 (ns)

**Table 6:** LD_50_ estimation using the up-and-down method for intraperitoneally injected mice at the dose of 10 mg/kg (i.e., 23.3 µmol/kg) of KM297 free base one minute after VX subcutaneous challenge.

KM297 Assumed LD_50_ = 1.1 ×LD_50_(VX)					
VX Dose (× LD_50_(VX))	0.91	1.0	1.1		All
Survival	1/1	1/3	0/1		2/5
LD_50_ = 1 × LD_50_(VX); 95% confidence interval [0; +∞[					

An LD_50_ value is initially assumed at the beginning of each experiment with sigma = 0.0414 (= log 1.1). The LD_50_ value was calculated using the AOT 425 Pgm software.

**Table 7:** LD_50_ estimation using the up-and-down method for untreated mice or injected intraperitoneally at the dose of 100 µmol/kg of 2-PAM or KM297 HCl and 62 µmol/kg of KM297 HCl one minute after paraoxon subcutaneous challenge. An LD_50_ value is initially assumed at the beginning of each experiment with sigma = 0.0414 (= log 1.1). The LD_50_ values were calculated using the AOT 425 Pgm software.

**No Treatment** Assumed LD_50_ = 964 µg/kg									
Paraoxon dose (µg/kg)	724		796		876				All
Survival	1/1		1/2		0/2				2/5
**LD_50_ = 796 µg/kg**; 95% Confidence Interval [283; 904]									
**2-PAM 100 µmol/kg** Assumed LD_50_ = 1.6 × LD_50_(Paraoxon)									
Paraoxon dose (× LD_50_(paraoxon))	1.45	1.60	1.76	1.94	2.13	2.34	2.58	2.83	All
Survival	1/1	1/1	1/1	1/1	1/1	1/1	2/3	0/2	8/11
**LD_50_ = 2.58 × LD_50_(paraoxon)**; 95% Confidence Interval [2.40; 2.92]									
**KM297 HCl 100 µmol/kg** Assumed LD_50_ = 1.2 × LD_50_(Paraoxon)									
Paraoxon dose (× LD_50_(paraoxon))	0.99		1.09		1.2				All
Survival	1/1		2/3		1/2				4/6
**LD_50_ = 1.18 × LD_50_(paraoxon)**; 95% Confidence Interval [0; +∞[									
**KM297 HCl 62 µmol/kg** Assumed LD_50_ = 2.13 × LD_50_(paraoxon)									
Paraoxon dose (× LD_50_(paraoxon))	1.76		1.94		2.13				All
Survival	1/1		2/3		0/2				3/6
**LD_50_ = 1.94 × LD_50_(paraoxon)**; 95% Confidence Interval [1.73; 2.25]									

**Table 8:** LD_50_ estimation of KM297 HCl using the up-and-down method for injected intraperitoneally mice. An LD_50_ value of 200 µmol/kg is initially assumed at the beginning of the experiment with sigma = 0.0176 (= log 1.5). The LD_50_ value was calculated using the AOT 425 Pgm software.

**No Treatment** Assumed LD_50_ = 200 µmol/kg					
KM297 HCl dose (µmol/kg)	133	200	300	450	All
Survival	1/1	2/2	1/2	0/1	4/6
**LD_50_ = 300 µmol/kg**; 95% Confidence Interval [206; 961]					
